# Phenotypical changes of satellite glial cells in a murine model of G_M1_‐gangliosidosis

**DOI:** 10.1111/jcmm.17113

**Published:** 2021-12-07

**Authors:** Bei Huang, Isabel Zdora, Nicole de Buhr, Deborah Eikelberg, Wolfgang Baumgärtner, Eva Leitzen

**Affiliations:** ^1^ Department of Pathology University of Veterinary Medicine Hannover Hannover Germany; ^2^ Center of Systems Neuroscience Hannover Germany; ^3^ Department of Biochemistry University of Veterinary Medicine Hannover Hannover Germany; ^4^ Research Center for Emerging Infections and Zoonoses (RIZ) University of Veterinary Medicine Hannover Hannover Germany

**Keywords:** dorsal root ganglia, G_M1_‐gangliosidosis, mouse, satellite glial cells, spinal ganglia

## Abstract

Satellite glial cells (SGCs) of dorsal root ganglia (DRG) react in response to various injuries in the nervous system. This study investigates reactive changes within SGCs in a murine model for G_M1_‐gangliosidosis (G_M1_). DRG of homozygous β‐galactosidase‐knockout mice and homozygous C57BL/6 wild‐type mice were investigated performing immunostaining on formalin‐fixed, paraffin‐embedded tissue. A marked upregulation of glial fibrillary acidic protein (GFAP), the progenitor marker nestin and Ki67 within SGCs of diseased mice, starting after 4 months at the earliest GFAP, along with intracytoplasmic accumulation of ganglioside within neurons and deterioration of clinical signs was identified. Interestingly, nestin‐positive SGCs were detected after 8 months only. No changes regarding inwardly rectifying potassium channel 4.1, 2, 3‐cyclic nucleotide 3‐phosphodiesterase, Sox2, doublecortin, periaxin and caspase3 were observed in SGCs. Iba1 was only detected in close vicinity of SGCs indicating infiltrating or tissue‐resident macrophages. These results indicate that SGCs of DRG show phenotypical changes during the course of G_M1_, characterized by GFAP upregulation, proliferation and expression of a neural progenitor marker at a late time point. This points towards an important role of SGCs during neurodegenerative disorders and supports that SGCs represent a multipotent glial precursor cell line with high plasticity and functionality.

## 1 INTRODUCTION

Satellite glial cells (SGCs) represent a unique cell population. They tightly surround neuronal cell bodies and thereby form discrete units in dorsal root ganglia (DRG), partly also referred to as spinal ganglia of the peripheral nervous system (PNS).^1^, ^2^ The sheath surrounding one neuronal soma is formed by multiple SGCs. Adjacent SGCs are connected with each other by adhesive and gap junctions and are separated from the vicinal perineural sheaths by small amounts of connective tissue.^3^, ^4^ The small physical distance between neurons and SGCs enables the latter to control the tissue environment, to modulate the activity of, as well as to communicate with neurons, very similar to the role of astrocytes in the central nervous system (CNS).^5^, ^6^ Interestingly, SGCs also share certain molecular markers with astrocytes, namely glutamine synthetase (GS)^7^ and the inwardly rectifying potassium channel (Kir) 4.1.^8^ Since these two markers neither are expressed in neurons nor in Schwann cells (SCs), they can be used as SGC‐specific markers for reliable identification of this cell population within murine DRG.^9^ Another shared feature of astrocytes and SGCs is represented by their ability to react to various noxious stimuli.^10^, ^11^, ^12^ Comparable to astrocytes within the CNS, SGCs are thought to proliferate and to convert into an activated state in response to injury, concomitant with an increased expression of glial fibrillary acidic protein (GFAP).^13^, ^14^ Therefore, GFAP is commonly used as a marker of murine SGC activation.^15^, ^16^, ^17^

Over the last years, increasing evidence suggests that SGCs not only respond to pathological conditions within the peripheral nerves (PN),^18^, ^19^ but also act as key modulators in chronic pain conditions.^20^, ^21^, ^22^, ^23^ This was further investigated using animal models of traumatic PN injury,^20^, ^23^, ^24^, ^25^ diabetic neuropathic pain,^5^, ^26^, ^27^ inflammatory pain as, for example in response to injection of the hindpaw with Complete Freund's Adjuvant^11^, ^28^, ^29^, ^30^ as well as herpes simplex infection.^31^, ^32^ Moreover, it is suggested that SGCs might possess a multipotent character with the ability to differentiate into sensory neurons as a consequence of nerve injury.^23^, ^33^ These results indicate that SGCs are not only functionally involved but might also represent a potential source of regenerative capacity in various pathological conditions.^23^, ^24^ These special features make SGCs extremely interesting research objects.

The knowledge of their exact role and behaviour in the context of storage diseases is scarce. Therefore, the aim of the present study was to characterize and investigate potential phenotypical changes in the expression pattern of SGCs in a lysosomal storage disease mouse model of G_M1_‐gangliosidosis. G_M1_‐gangliosidosis in humans is associated with degenerative changes within CNS and PNS.^34^, ^35^, ^36^ The clinical disease is divided into three types, according to the age of onset including an infantile type with early onset and rapid clinical deterioration, a late infantile/juvenile type with later onset and prolonged progression as well as an adult type.^37^ It is caused by an accumulation of G_M1_‐ganglioside due to a deficiency of β‐galactosidase (GLB1).^37^, ^38^, ^39^ Consequently, G_M1_‐gangliosides and related glycoconjugates are deposited in several tissues but especially within neurons.^37^ This leads to distension with subsequent death of neurons within both, CNS and PNS.^37^, ^40^, ^41^ Studying murine models for G_M1_‐gangliosidosis, it was observed that Glb1‐deficient mice, despite increasing accumulations of G_M1_‐ganglioside, did not show clinical abnormalities up to the age of 4–5 months.^35^, ^42^

The present study investigates the phenotypical changes of SGCs within the DRG of C57BL/6 wild‐type (Glb1^+/+^; WT) and homozygous β‐galactosidase‐knockout (Glb1^−/−^) mice. Because of the close interrelationship between neuronal somata and SGCs as well as the intense communication between both cell types, it was hypothesized that SGCs of affected mice will show phenotypical changes during the course of disease. A better understanding of the reaction pattern and the potential involvement of SGCs during the course of G_M1_‐gangliosidosis will increase our knowledge about the nature and the potential of this unique cell population.

## 2 MATERIALS AND METHODS

### 2.1 Animals and clinical investigation

Dorsal root ganglia of homozygous Glb1 knockout and WT mice were obtained from previous experiments.^40^ In brief, Glb1^−/−^ mice were generated via insertion of a lacZ gene fragment of 636 base pairs into exon 15 of the *Glb1* gene of murine C57BL/6 oocytes. Genotyping of mice was achieved using conventional polymerase chain reaction and gel electrophoresis.^40^ Both Glb1^−/−^ and WT mice were bred and housed in parallel, as described previously.^40^ Mice were examined regularly assessing clinical parameters like appearance/posture, behaviour/activity and gait. Furthermore, animals were screened for neurological deficiencies using the parachute reflex test and grid‐walking test (horizontal wire netting).^40^ At 2, 4, 6 and 8 months of age, 6 WT and Glb1^−/−^ mice were euthanized, and DRG were removed at the height of the cervical vertebral column. Tissue was routinely fixed in 10% formalin and embedded in paraffin wax.

### 2.2 Tissue processing and evaluation

Formalin‐fixed and paraffin‐embedded tissue samples were used for immunofluorescence (IF) analysis. All tissue samples were cut into approximately 4 µm thick sections on a microtome and subsequently mounted on SuperFrost‐Plus^®^ slides (Thermo Fisher Scientific Inc., Fisher Scientific GmbH). IF staining was performed as previously described.^9^ Briefly, deparaffinization and rehydration were performed following standard procedures using xylene and graded alcohols. Sections were blocked in 20% goat serum in phosphate buffered saline (PBS) containing 1% bovine serum albumin (BSA) and 0.1% Triton‐X (Triton^®^ X‐100, Merck millipore, Merck KGaA) after antigen retrieval in citrate buffer (pH = 6) for 20 min in a microwave. Primary antibodies (for details see Table 1) against caspase 3 (diluted 1:100), 2,3‐cyclic nucleotide 3‐phosphodiesterase (CNPase; diluted 1:500), GFAP (diluted 1:400), GS (diluted 1:2000 for polyclonal rabbit; 1:400 for monoclonal mouse), Iba1 (diluted 1:400), inwardly rectifying potassium channel Kir4.1 (diluted 1:2000), Ki67 (diluted 1:500), nestin (diluted 1:250) and periaxin (diluted 1:500) were incubated on sections at 4°C overnight. Rabbit IgG (02–6102, Invitrogen, Thermo Fisher Scientific) and normal mouse serum (Biologo; Dr. Hartmut Schultheis eK; Immunologische Produkte, Kronshagen; CL8100) were used as negative controls accordingly. For visualization, goat anti‐rabbit Cy2 (diluted 1:200; 111–225–144, Jackson ImmunoResearch Europe Ltd) and goat anti‐mouse Alexa fluor 488 (diluted 1:200; 115–545–003, Jackson ImmunoResearch Europe Ltd) were incubated at room temperature for 1 h. Nuclei were stained with bisbenzimidin (diluted in Aqua bidestillata; bisBenzimide H 33258, Merck KGaA) followed by mounting sections with fluorescence mounting medium (Dako North America Inc.).

**TABLE 1:** Primary antibodies used for immunofluorescence (IF) and immunohistochemistry (IHC)

Primary antibody specificity	Clonality	Source	Dilution
Caspase 3	mc rabbit	9661s, Cell Signaling Technology Inc., Danvers, MA, USA	1:100 (IF)
CNPase	mc mouse	MAB326, clone 11‐5B, Sigma‐Aldrich, Merck KGaA, Darmstadt, Germany	1:100 (IF)
Doublecortin	mc mouse	sc−271390, Santa Cruz Biotechnology, Inc., Dallas, TX, USA	1:100 (IHC)
GFAP	pc rabbit	Z0334, Dako North America Inc., Carpinteria, CA, USA	1:400 (IF)
GM1‐1	mc mouse	SH30349, Developmental Studies Hybridoma Bank (DSHB); GM1‐1 was deposited to the DSHB by Schnaar, R.L., University of Iowa, Iowa City, IA, USA	1:26 (IF)
GS	pc rabbit	PA5‐28940, Invitrogen, Thermo Fisher Scientific, Waltham, MA, USA	1:2000 (IF)
GS	mc mouse	GT1055, Invitrogen, Thermo Fisher Scientific, Waltham, MA, USA	1:400 (IF)
Iba1	pc goat	011–27991, FUJIFILM Wako Pure Chemical Corporation, Osaka, Japan	1:400 (IF)
Kir 4.1	pc rabbit	APC−035, Alomone laboratories Ltd, Jerusalem, Israel	1:2000 (IF)
Ki67	pc rabbit	Ab15580, Abcam, Cambridge, UK	1:500 (IF)
Nestin	pc rabbit	AP 07829PU‐N, OriGene Technologies, Rockville, MD, USA	1:250 (IF)
NG2	pc rabbit	AB5320, Sigma‐Aldrich, Merck KGaA	1:800 (IHC)
Periaxin	pc rabbit	HPA001868‐100UL, Sigma‐Aldrich, Merck KGaA, Darmstadt, Germany	1:500 (IF)
Sox2	mc rabbit	3579S, Cell Signaling Technology Inc., Danvers, MA; USA	1:50 (IHC)

Abbreviations: CNPase, 2’,3'‐cyclic nucleotide 3'‐phosphodiesterase; GFAP, glial fibrillary acidic protein; GS, glutamine synthetase; Iba1, ionized calcium‐binding adapter molecule 1; Kir 4.1, inwardly rectifying potassium channel 4.1; mc, monoclonal; NG2, neural/glial antigen 2; pc, polyclonal; Sox2, sex determining region Y‐box 2.

Furthermore, storage material within sensory neurons of the DRG was visualized performing a luxol fast blue (lipid stain; LFB) and cresyl violet stain (Nissl substance stain) as well as immunofluorescence with an anti‐GM1 antibody. The antibody targeting GM1 (diluted 1:26) was deposited to the Developmental Studies Hybridoma Bank (DSHB) by Schnaar, R.L. (DSHB Hybridoma Product GM1‐1). For selected antigens, immunohistochemistry (IHC) using the avidin‐biotin‐peroxidase complex (ABC) method was performed according to previous studies.^9^ Primary antibodies against doublecortin (diluted 1:100), neural/glial antigen 2 (NG2; diluted 1:800) and Sox2 (diluted 1:50) were incubated overnight in PBS and 1% bovine serum albumin (BSA) at 4°C. Staining was visualized with 3,3‐diaminobenzidine tetrahydrochloride (DAB, 0.05%, Sigma‐Aldrich Chemie GmbH) with addition of 0.03% H2O2 and counterstaining with Mayer's haematoxylin (Roth C. GmbH & Co KG), dehydrated and mounted with ROTI^®^ Histokitt II (Roth C. GmbH & Co KG).

For transmission electron microscopy, selected DRG were fixed in 5% glutaraldehyde in cacodylate buffer and further processed as described previously.^40^ Accumulated storage material within neurons was visualized using a transmission electron microscope (Zeiss EM 10C electron microscope; Zeiss).

For quantification of immunostainings, pictures of all DRG analysed were taken with a Keyence BZ 9000 fluorescent microscope (Keyence) with Nikon Plan Apo λ objectives (Nikon Europe BV). Afterwards, images were analysed by manual counting of all visible neurons (total number of neurons) and of neurons surrounded by immunopositive SGCs (total number of neurons surrounded by positive SGCs). The percentage of neurons surrounded by positive SGCs per DRG for each marker was calculated as the ratio of total number of neurons surrounded by positive SGCs divided by total number of neurons. A minimum of 5 up to 17 DRG pooled from three animals for each condition were used for comparison.

Selected markers (GFAP, Iba1, Ki67 and nestin) were double labelled with GS and analysed in confocal microscopy in order to reassure their localization within the DRG and for improved visualization. Pictures were captured with a Leica TCS SP5 AOBS confocal inverted‐base fluorescence microscope (Leica Microsystems) with a HCX PL APO lambda blue 63.0 x 1.40 oil immersion objective. The laser settings were adjusted in conformity with the appropriate negative controls. Additionally, z‐stacks were generated and analysed with LAS X 3D version 3.1.0 software from Leica. Moreover, representative immunofluorescence double labelling of GS and GM1 as well as nestin and GM1 was performed to visualize storage material within DRG neurons surrounded by SGCs positive for nestin. Luxol fast blue cresyl violet‐stained slides were digitalized with a DP72 camera (Olympus) mounted on a BX51 microscope (Olympus). Pictures were taken using a 60x objective with oil immersion.

### 2.3 Statistical analysis

Statistical analysis was performed using SPSS for Windows (version 27; IBM^®^ SPSS^®^ Statistics, SPSS Inc.). Differences between groups at different time points were analysed via Mann‐Whitney U tests. Statistical significance was accepted at a *p*‐value of <0.05. For statistical evaluation of immunostainings, initially only 2‐ and 8‐month‐old animals were investigated for significant alterations between affected and non‐affected animals (CNPase, doublecortin, GFAP, Ki67, nestin, periaxin, Sox2) and/or in between time points (Kir 4.1). Where significant changes were detected, 4‐ and 6‐month‐old animals were evaluated accordingly to further investigate the chronological sequence of changes. For evaluation of clinical data matching the investigated tissue specimens, the number of mice included in the statistical analysis was *n* = 18 (2 months of age), *n* = 17 (4 months of age), *n* = 12 (6 months of age) and *n* = 6 (8 months of age) for Glb1^−/−^ mice, and *n* = 16 (2 months of age), *n* = 12 (4 months of age), *n* = 12 (6 months of age) and *n* = 5 (8 months of age) for WT mice.

### 2.4 Ethics

All animal experiments were conducted in accordance with the German Animal Welfare Law and were approved by local authorities (Niedersächsisches Landesamt für Verbraucherschutz und Lebensmittelsicherheit (LAVES), Oldenburg, Germany, permission number: 33.9–42502–04‐14/1532).

## 3 RESULTS

### 3.1 Clinical deterioration in Glb1^−/−^ mice starts at 4 months of age

**FIGURE 1:**
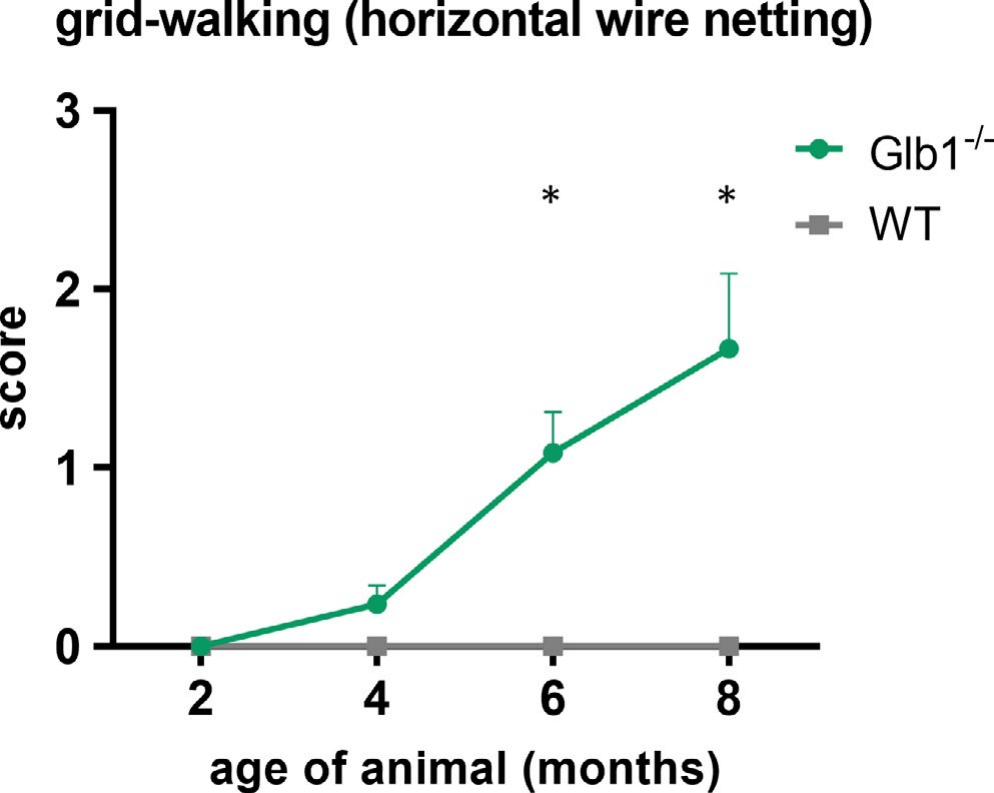
Evaluation of horizontal wire netting test (grid‐walking test) of Glb1^−/−^ and wild‐type (WT) mice over the course of 2–8 months of age (x‐axis). Mice were scored with a 0–3 point system (y‐axis) [0 = mice do not step into the mesh circuit; 1 = 21–30 s until stepping into the mesh circuit; 2 = 11–20 s until stepping into the mesh circuit; and 3 = 0–10 s until stepping into the mesh circuit]. Elevated scores in Glb1^−/−^ mice were first detected at the age of 4 months. Significantly elevated scores in Glb1^−/−^ mice were observed at the age of 6 and 8 months. Graphs display mean values including standard error of the mean. Significant differences detected by Mann‐Whitney U tests are indicated by asterisks (*p < 0.05)

**FIGURE 2:**
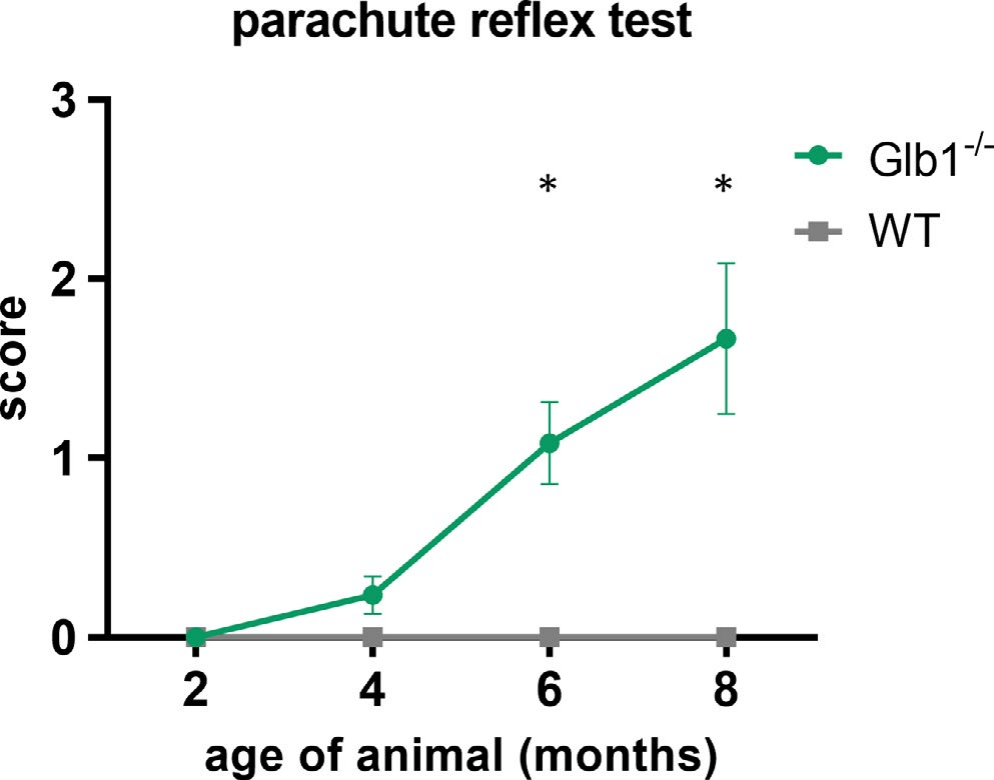
Evaluation of parachute reflex test of Glb1^−/−^ and wild‐type (WT) mice over the course of 2 to 8 months of age (x‐axis). Mice were scored using a 0–3 point system (y‐axis) [0 = extension and abduction of the hind limbs and extension of the knee; 1 = mildly delayed reaction and intermitting extension of the knee; 2 = moderately delayed reaction, flexion and abduction of the hind limbs and slow movement; and 3 = no reaction, continuous flexion and adduction of the hind limbs]. Elevated scores in Glb1^−/−^ mice were first detected at the age of 4 months. Significantly elevated scores in Glb1^−/−^ mice were observed at the age of 6 and 8 months. Graphs display mean values including standard error of the mean. Significant differences detected by Mann‐Whitney U tests are indicated by asterisks (*p < 0.05)

Elevated scores indicating an onset of neurological signs in Glb1^−/−^ mice could be already observed at the age of 4 months in both tests applied (Figures 1 and 2). Significant neurological dysfunction was observed in Glb1^−/−^ mice starting at 6 months of age. These results match previous investigations, stating an onset of clinical signs around the age of approximately 4 months^35^, ^42^ in G_M1_‐gangliosidosis and an ‘increasing neurological disorder starting at the age of 3.5–4 months’ in Glb1^−/−^ mice.^40^ Based on these findings, the question arose whether clinical impairment was linked to morphological changes in DRG neurons as well as an altered expression pattern of SGCs.

### 3.2 Accumulated storage material is visible in sensory neurons of dorsal root ganglia but not satellite glial cells

**FIGURE 3:**
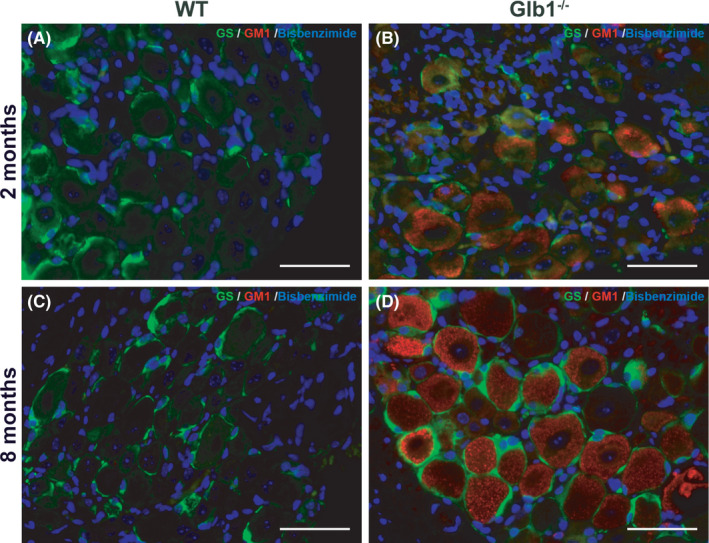
Representative images of immunofluorescence double staining of murine dorsal root ganglia (DRG) of 8‐month‐old Glb1^−/−^ and wild‐type (WT) mice with the satellite glial cell (SGC)‐specific marker glutamine synthetase (GS; green) and GM1 (red). Nuclei are counterstained with bisbenzimide (blue). (A, C) DRG of WT mice show no accumulation of GM1 material. (B, D) Sensory neurons of Glb1^−/−^ mice show accumulation of GM1‐positive lysosomal storage material, whereas surrounding, GS‐positive SGCs do not display any immunoreaction for GM1. Scale bar, 50 μm. Nuclei are counterstained with bisbenzimide (blue)

Electron microscopic images of a DRG from an 8‐month‐old Glb1^−/−^ mouse clearly display that there is accumulation of lamellar, lysosomal storage material in the cytoplasm of sensory neurons. Adjacent SGCs do not accumulate storage material (Figure S1). Furthermore, the luxol fast blue and cresyl violet stain illustrates the cytoplasmic accumulation of DRG neurons by LFB‐positive, blue‐stained, lipid‐rich lysosomal storage material and marginalized Nissl substance (Figure S2). Immunofluorescence double labelling of murine DRG with the SGC‐specific marker GS and an anti‐GM1 antibody further corroborate the perception that SGCs do not accumulate GM1. GS‐positive SGCs show no co‐labelling with GM1 (Figure 3). Marked GM1‐immunoreactivity is visible in sensory neurons only.

### 3.3 Kir 4.1 expression in Glb1^−/−^ mice remains constant during G_M1_‐gangliosidosis

**FIGURE 4:**
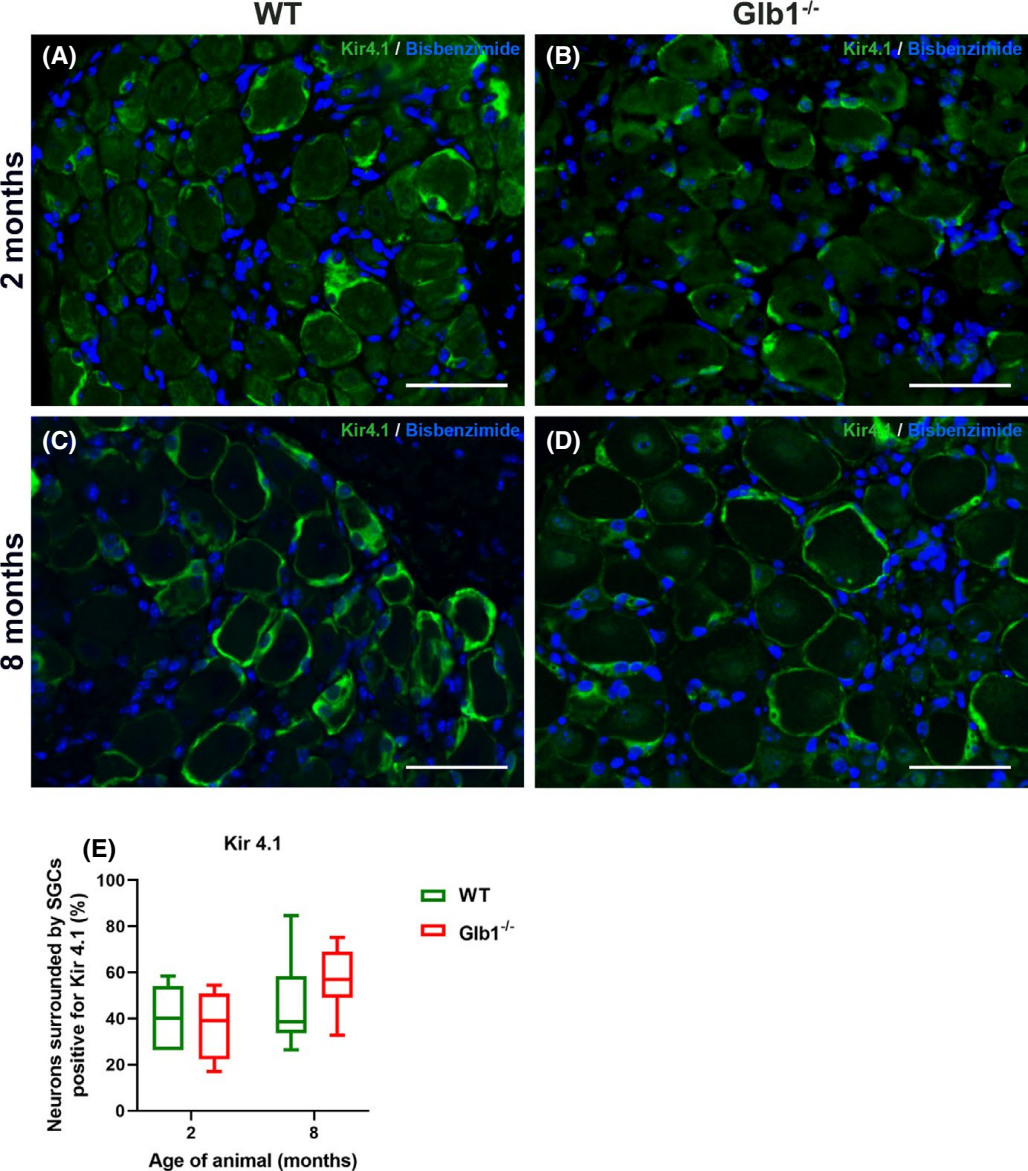
Representative images of immunofluorescence staining of murine dorsal root ganglia (DRG) with inwardly rectifying potassium channel Kir 4.1 (green) including statistical analysis. Nuclei are counterstained with bisbenzimide (blue). (A‐D) Pictures show a high percentage of satellite glial cells (SGCs) immunopositive for the SGC‐specific marker Kir 4.1, tightly surrounding neurons at all time points investigated (2–8 months). Scale bar: 50 μm. (E) Quantification of neurons surrounded with Kir4.1‐positive SGCs indicated no significant changes of Kir 4.1 expression in Glb1^−/−^ mice between 2 (*n* = 5 DRG for Glb1^−/−^, *n* = 10 DRG for WT) and 8 (*n* = 15 DRG for Glb1^−/−^, *n* = 15 DRG for WT) months of age. Graphs display box and whisker plots

Kir 4.1 is a suitable marker for the detection of murine SGCs and stains a high percentage of these cells per DRG.^9^ Kir 4.1 is also reported to be downregulated in various pathological conditions.^43^, ^44^ However, within the present study, no significant changes of Kir 4.1 expression could be detected in Glb1^−/−^ mice between 2 and 8 months of age (Figure 4).

### 3.4 Satellite glial cells express GFAP in Glb1^−/−^ mice

**FIGURE 5:**
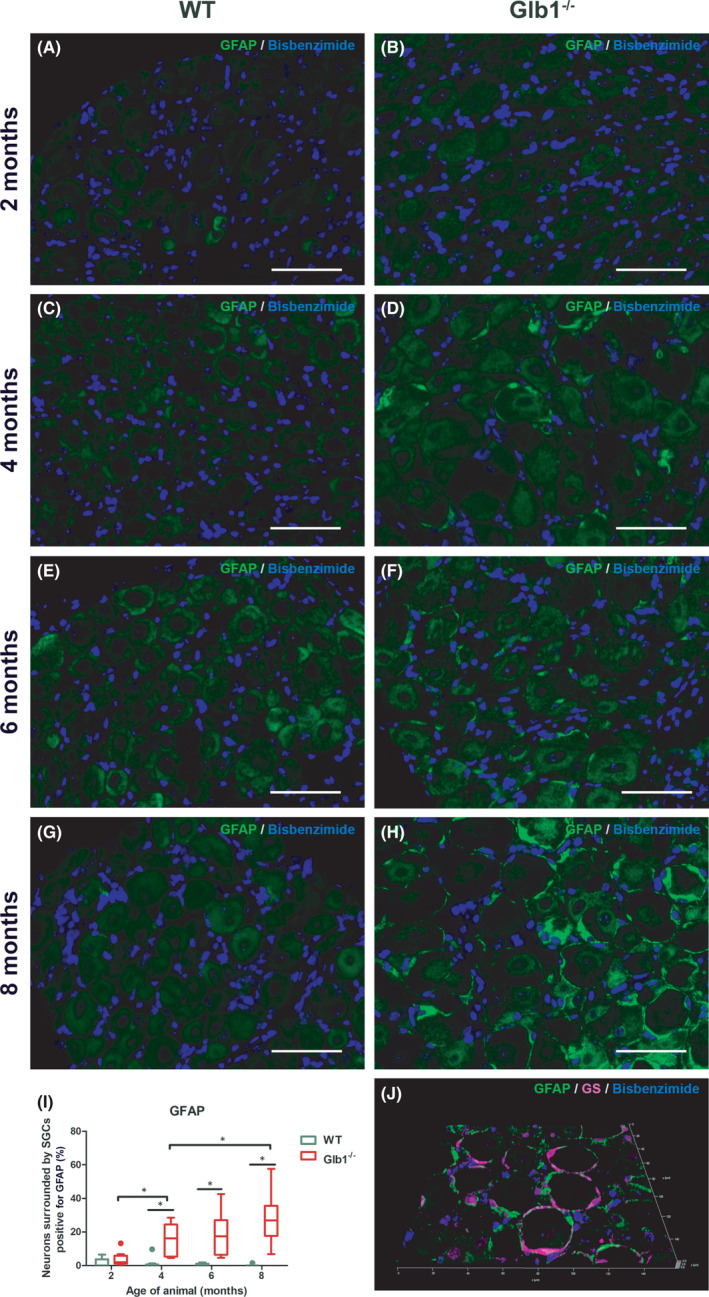
Representative images of immunofluorescence staining of murine dorsal root ganglia (DRG) with glial fibrillary acidic protein (GFAP) including statistical analysis. (A, C, E, G) No GFAP expression was detected in SGCs of wild‐type (WT) mice at any investigated time point (2–8 months). (B, D, F, H) Glb1^−/−^ mice showed increasing GFAP immunoreactivity (GFAP, green) over the observation period, indicating satellite glial cell (SGC) activation. Scale bar, 50 μm. (I) Quantification of neurons surrounded by immunopositive SGCs, with significant differences between Glb1‐/‐ and WT mice at 4, 6 and 8 months. Graphs display box and whisker plots. Significant differences as detected by Mann‐Whitney U test are indicated by asterisks (* *p* < 0.05; *n* = 3 mice; 2 months: *n* = 8 DRG for Glb1^−/−^, *n* = 13 DRG for WT; 4 months: *n* = 9 DRG for Glb1^−/−^, *n* = 10 DRG for WT; 6 months: *n* = 11 DRG for Glb1^−/−^, *n* = 16 DRG for WT; 8 months: *n* = 15 DRG for Glb1^−/−^, *n* = 14 DRG for WT;). (J) 3D reconstructed confocal laser image of a representative DRG of a Glb1^−/−^ mouse at 8 months double labeled with GFAP (green) and the SGC‐specific marker glutamine synthetase (GS; magenta). 48 z‐stack frames (5.92 μm total size; approx. 0.13 μm steps)

Previous studies have shown that SGCs show activation by increasing the expression of GFAP after injury.^20^, ^45^, ^46^ G_M1_‐gangliosidosis is a lysosomal storage disorder that manifests as a progressive neurological disease. Accordingly, axonal and neuronal damage increase within Glb1^−/−^ mice during disease progression.^40^ DRG sections were stained using an anti‐GFAP antibody to determine, whether SGCs of Glb1^−/−^ mice express GFAP during G_M1_‐gangliosidosis. IF analysis revealed that expression of GFAP is almost absent in WT mice with only very few positive SGCs (Figure 5). However, GFAP expression is markedly increased in SGCs of Glb1^−/−^ mice. A significant increase compared to WT mice could already be detected starting at 4 months of age. The number of neurons surrounded by positive SGCs showed a consistently increasing trend in the course of disease with significantly higher numbers comparing 2 and 4 as well as 4 and 8 months. These data indicate that SGCs increasingly upregulate GFAP.

### 3.5 Satellite glial cells show proliferation but no increased apoptosis in Glb1^−/−^ mice

**FIGURE 6:**
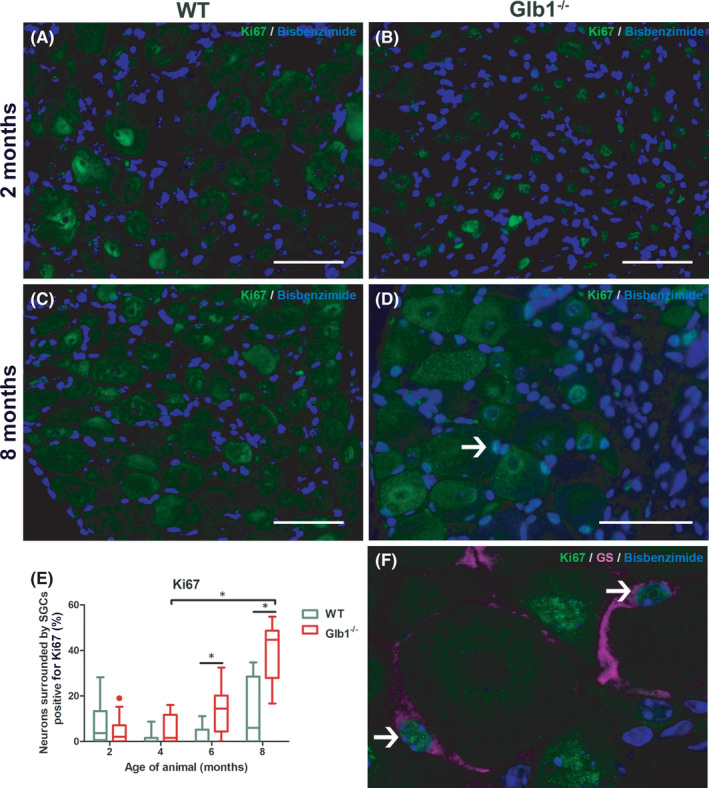
Representative images of immunofluorescence staining of murine dorsal root ganglia (DRG) with Ki67 indicating proliferation of satellite glial cells (SGCs) including statistical analysis. (A, C) Wild‐type mice exhibit a low basal proliferation rate in DRG. (B, D) Glb1^−/−^ mice show increasing numbers of Ki67‐positive SGCs between 2 and 8 months. Nuclei of SGCs display Ki67‐immunoreactivity (D; arrow). Scale bar, 50 μm. (E) Quantification of neurons surrounded by immunopositive SGCs. Graphs display box and whisker plots. Significant differences as detected by Mann‐Whitney U test are indicated by asterisks (*p < 0.05; *n* = 3 mice; 2 months: *n* = 11 DRG for Glb1^−/−^, *n* = 16 DRG for WT; 4 months: *n* = 7 DRG for Glb1^−/−^, *n* = 5 DRG for WT; 6 months: *n* = 8 DRG for Glb1^−/−^, *n* = 8 DRG for WT; 8 months: *n* = 17 DRG for Glb1^−/−^, *n* = 15 DRG for WT). (F) Confocal laser image of a representative DRG of a Glb1^−/−^ mouse at 8 months double labeled with Ki67 (green; arrows) and the SGC‐specific marker glutamine synthetase (GS; magenta)

It has also been demonstrated that SGCs show proliferation after injury.^23^, ^47^, ^48^ In order to further characterize the response of murine SGCs in Glb1^−/−^ mice, DRG sections were labelled with an anti‐Ki67 antibody and analysed by IF. Ki67‐positive nuclei were detected in SGCs and SCs. Significant differences between affected and non‐affected animals could be detected starting at 6 months with significantly increasing numbers until the end of the investigation period (8 months; Figure 6). Only a small portion of cells showed immunopositivity for Ki67 in WT mice. These findings indicate that G_M1_‐gangliosidosis induces an accelerated proliferation of SGCs. Occasionally, positive signals were detected within nuclei of DRG neurons in both, Glb1^−/−^ and WT mice. Within the present study, however, quantification of Ki67‐positive neurons did not reveal significant differences between Glb1^−/−^ and WT mice. Furthermore, using an anti‐caspase 3 antibody, there was no evidence for increased apoptosis of SGCs within DRG of affected mice.

### 3.6 Satellite glial cells show expression of nestin in Glb1^−/−^ mice at the end of the investigation period

**FIGURE 7:**
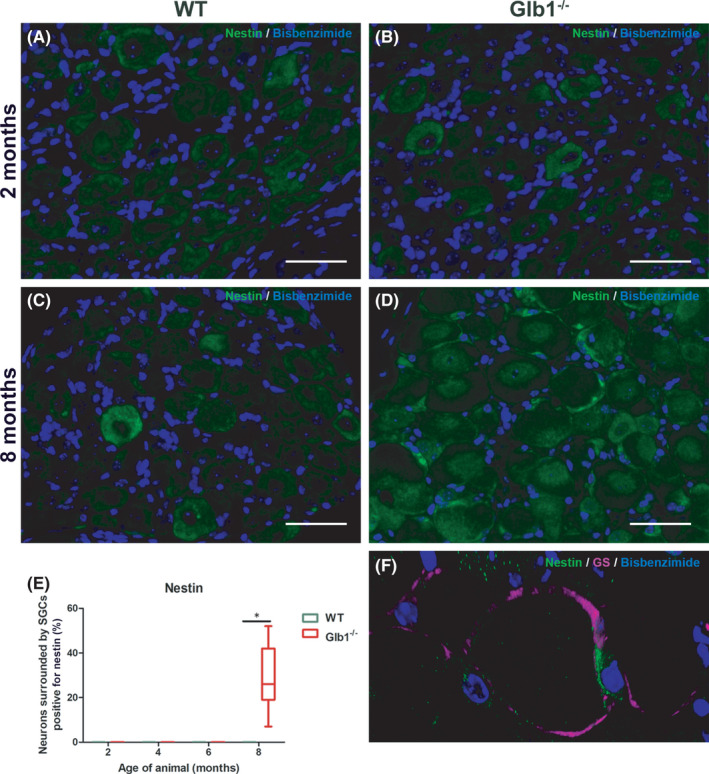
Representative images of immunofluorescence staining of murine dorsal root ganglia (DRG) with nestin, a neural progenitor cell marker, including statistical analysis. (A, C) No nestin expression was detected in wild‐type mice at any time point. (B, D) Glb1^−/−^ mice showed nestin immunoreactivity in satellite glial cells (SGCs) only at the end of the investigation period at 8 months of age. Scale bar, 50 μm. (E) Quantification of neurons surrounded by immunopositive SGCs. Graphs display box and whisker plots. Significant differences as detected by Mann‐Whitney U test are indicated by asterisks (*p < 0.05) (*n* = 3 mice; 2 months: *n* = 9 DRG for Glb1^−/−^, *n* = 12 DRG for WT; 4 months: *n* = 9 DRG for Glb1^−/−^, *n* = 8 DRG for WT; 6 months: *n* = 12 DRG for Glb1^−/−^, *n* = 13 DRG for WT; 8 months: *n* = 17 DRG for Glb1^−/−^, *n* = 14 DRG for WT). (F) 3D reconstructed confocal laser image of a representative DRG of a Glb1^−/−^ mouse at 8 months double labelled with nestin (green) and SGC‐specific marker glutamine synthetase (GS; magenta). 43 z‐stack frames (5.29 μm total size; approx. 0.13 μm steps)

Nestin is an intermediate filament that is commonly expressed in neural progenitor cells and gets downregulated during cellular differentiation.^49^ Interestingly, it has been reported that SGCs show nestin expression during embryonic stages and that nestin expression might be re‐activated following injury.^23^, ^50^ Within the present study, the occurrence of nestin‐positive SGCs was detected in Glb1^−/−^ mice at the end of the investigation period (Figure 7; Figure S3). Nestin‐positive SGCs surround sensory neurons, which show a positive immunoreaction for GM1 (Figure S3).

### 3.7 Murine satellite glial cells show no expression of Sox2 and doublecortin

In search for the potential upregulation and/or expression of further progenitor markers, DRG were investigated using anti‐Sox2, anti‐NG2 and anti‐doublecortin antibodies. No immunoreactivity for either Sox2 or doublecortin (Figure S4) was found in SGCs of Glb1^−/−^ and WT mice. Moreover, DRG neurons did not show any immunoreactivity for doublecortin. As experienced previously,^9^ evaluation of NG2 staining on FFPE tissue did not reveal a distinct and evaluable staining pattern.

### 3.8 Murine satellite glial cells show no expression of markers for myelination

To further determine a potential re‐myelinating capacity of SGCs, DRG were investigated using anti‐CNPase and anti‐periaxin antibodies. These two markers represent myelin markers of the CNS (CNPase) and PNS (periaxin) respectively. SGCs of neither affected nor unaffected animals showed immunopositivity for CNPase and/or periaxin.

## 4 DISCUSSION

G_M1_‐gangliosidosis is a lysosomal storage disease caused by a predominantly intraneuronal accumulation of G_M1_‐gangliosides, leading to a progressive neurodegenerative disorder.^38^ Although CNS disease represents the most prominent feature of most lysosomal storage disorders, the PNS has been reported to be involved, too. In a previous study using Glb1^−/−^ mice, DRG neurons showed accumulation of lamellar and partly concentrically arranged material within distended lysosomes, whereas adjacent SGCs and SCs did not show any signs of intracellular storage of gangliosides.^40^ However, the exact role of SGCs in lysosomal storage disorders has not been investigated yet. In the present study, the aim was to investigate whether SGCs in murine DRG react to neuronal accumulation of G_M1_‐gangliosides in Glb1^−/−^ mice compared to WT mice. Therefore, a representative set of markers based on previous own studies^9^, ^51^ was chosen for analysis.

### 4.1 Kir 4.1 expression is not downregulated during G_M1_‐gangliosidosis

Previous studies suggested that the SGC expression of Kir 4.1 is downregulated in response to PN axotomy and during chronic pain conditions.^43^, ^44^ However, a decrease in Kir 4.1 expression could not be detected in Glb1^−/−^ mice at the latest time point (8 months; Figure 4).

Nevertheless, murine SGCs show phenotypical changes during G_M1_‐gangliosidosis in a time‐dependent manner. Interestingly, the first alteration determined was a detection of GFAP‐positive SGCs in G_M1_^−/−^ mice at 4 months. With the disease advancing, proliferation, detected by an increased amount of Ki67‐positive SGCs got evident starting at 6 months. Finally, at the age of 8 months, SGCs started to express the stem cell marker nestin.

### 4.2 Satellite glial cells upregulate GFAP concomitant to progression of G_M1_‐gangliosidosis

Glial fibrillary acidic protein is an intermediate filament that is mainly expressed in astrocytes and is upregulated following brain injury with increasing GFAP levels frequently being used as a marker for CNS astrogliosis.^52^, ^53^ It is believed that the increased expression of GFAP in astrocytes is involved in the formation of elongated and thickened processes and represents a prominent feature following traumatic and degenerative events within the CNS.^54^ A reactive astrogliosis of the CNS is also present in murine and canine models of G_M1_‐gangliosidosis as well as during the course of the human disease, especially within areas exhibiting neuronal vacuolation, neuronal death and demyelination.^37^, ^55^, ^56^

Although separated within distinct compartments of the nervous system, astrocytes and SGCs show various similarities with regard to cellular physiology, signalling properties and function, especially during pathological conditions.^57^ In previous studies, it was confirmed that the majority of canine SGCs show immunopositivity for GFAP, even in adult, healthy animals. However, SGCs of healthy, adult mice did not show detectable levels of GFAP using IF.^9^, ^51^ Nevertheless, comparable to astrocytes within the CNS, there is evidence that murine SGCs upregulate GFAP expression in several pathological conditions, including systemic inflammation^29^ and several neuropathic pain models.^13^, ^20^, ^45^, ^58^

An increased number of GFAP‐positive murine SGCs in Glb1^−/−^ mice could be detected starting from the age of 4 months. Moreover, the number of neurons encircled by GFAP‐positive SGCs increased with disease duration until 8 months (Figure 5). Interestingly, the time point of GFAP upregulation within SGCs corresponds with the reported onset of apparent clinical signs in Glb1^−/−^ mice.^35^, ^40^, ^42^ It seems that within both compartments, CNS and PNS, glial cells react to the accumulation of gangliosides in neurons.^40^ It has been hypothesized that changes in the neuronal activity may induce alterations in the GFAP expression of astrocytes, a theory that might also be extrapolated to SGCs, indicating a crucial role of GFAP in glia cell‐neuron interaction.^59^ This is of special interest since previous studies detected a significantly larger cell capacitance in swollen neurons of the medial nucleus of the trapezoid body within the mouse model used within the present study.^40^

### 4.3 Murine satellite glial cells exhibit an increased proliferation rate at the age of 6 months

As stated before, SGCs tightly envelop neurons and provide support. They perform similar functions in the periphery as astrocytes in the CNS. Neuronal injury within the CNS is frequently associated with the development of a reactive astrogliosis, which is not only characterized by upregulation of GFAP but also by proliferation of astrocytes.^54^ In line with this, previous studies have shown that activation of SGCs also includes both upregulation of GFAP and proliferation.^11^, ^19^, ^20^, ^31^, ^45^, ^46^, ^60^ In general, SGCs retain the ability of cell division during adult life under physiological conditions, although the turnover rate of this cell population is slow under normal conditions, like seen in the WT group of the present study.^19^ Increased proliferation of murine and rat SGCs, however, was found in various pathological conditions including skin scarification,^19^ dental injury,^11^ monoarthritis^61^ and following herpes virus infection.^31^

In the present study, detection of Ki67 antigen via IF was used to quantify SGC proliferation. The number of neurons encircled by Ki67‐positive SGCs was significantly elevated at 6 and 8 months in Glb1^−/−^ mice compared to WT mice (Figure 6). Despite this proliferation, there was no indication of enhanced apoptosis of SGCs as tested using an anti‐caspase 3 antibody. These results suggest that the proliferation of SGCs does not represent a simple countermeasure against an increased SGC loss during disease progression. Proliferation might therefore represent an attempt of SGCs to maintain homeostasis and structural integrity within the DRG as well as to secure neuronal survival, comparable to glial cell proliferation within the injured CNS.^62^

Interestingly, previous studies indicate that GFAP expression not only promotes proliferation within CNS astrocytes^52^ but also relates to proliferation of SCs of the PNS and subsequent regeneration.^60^ This may point to a possible link between GFAP expression in SGCs and the following increased proliferation rate of SGCs.

### 4.4 Glb1^−/−^ mice upregulate nestin in satellite glial cells at the end of the investigation period

The intermediate filament nestin has been widely accepted as a marker for multipotent stem cells and progenitor cells in various tissue, for example muscle,^63^ hair follicle sheath,^64^ pancreas^65^ and teeth.^66^ Importantly, nestin is also a marker for neuronal and glial cells, together with their shared progenitor cells.^67^ However, nestin expression is temporary in most of these cells and downregulated during cell differentiation.^68^ In adults, the expression of nestin is mainly restricted to stem cell niches like the subventricular zone as well as the hippocampus in the CNS.^69^ However, an increased number of nestin‐positive cells can be seen in response to several pathologic conditions affecting the CNS, as for example inflammation, ischaemia and epilepsy.^67^ Several studies suggest that nestin is re‐expressed and upregulated in activated astrocytes,^70^, ^71^ as well as in SGCs following nerve injury.^72^, ^73^

The present study shows that adult murine SGCs in Glb1^−/−^ mice show expression of nestin at the end of the investigation period (8 months) (Figure 7; Figure S3). Within WT mice, no nestin‐positive SGCs could be detected. This result matches previous data of healthy adult murine and canine DRG lacking SGC immunoreactivity for nestin.^9^ These data indicate an upregulation of nestin in SGCs of Glb1^−/−^ mice in the context of ganglioside accumulation within neurons but not SGCs at the age of 8 months. Moreover, it might indicate a conversion towards a more immature phenotype with enhanced plasticity and mobility.^70^, ^74^ A dormant stem cell character might include the possibility to use SGCs as a source of regenerative capacity during various diseases.

### 4.5 Murine satellite glial cells do not upregulate expression of precursor markers Sox2 and doublecortin

In this study, murine SGCs of neither Glb1^−/−^ nor WT mice showed any immunoreaction for Sox2 or doublecortin. Sox2 is a transcription factor expressed by neural/glial precursors,^51^ and doublecortin is expressed by neuronal precursors.^52^ The lack of Sox2 expression in adult murine SGCs correlates with previous studies.^9^ Doublecortin as a neuronal precursor marker in adult neurogenesis^75^ can also be expressed in sensory neurons of adult murine DRG.^76^ However, in this study, no immunoreaction for doublecortin was observed in sensory neurons of DRG (Figure S4). Furthermore, NG2, a well‐established marker for oligodendrocyte precursor cells which is expressed in a subgroup of SGCs in C57BL/6 wild‐type mice,^9^ was tested. As experienced previously,^9^ NG2 is very sensitive towards formalin fixation with lack of a distinct and evaluable staining pattern on FFPE tissue. Therefore, this marker was determined as non‐suitable for evaluation of the available tissue.

### 4.6 Murine satellite glial cells do not express markers of myelination

Myelination of nerve fibres is crucial for nerve conduction. Oligodendrocytes and SCs within the CNS and PNS, respectively, are specialized glial cells that are in charge of myelin production.^77^ CNPase is a myelin‐associated enzyme mainly found in oligodendrocytes that is mandatory for physiological function of the axon‐myelin unit.^78^, ^79^ Within the PNS, periaxin represents a SC‐specific protein of non‐compact myelin sheaths.^80^, ^81^ A loss of proper myelination represents a key feature of several devastating diseases like, for example multiple sclerosis,^82^ amyotrophic lateral sclerosis^83^, ^84^ or Charcot‐Marie‐Tooth disease.^85^

Previous studies have shown that canine SGCs of healthy, adult dogs express CNPase; however, this does not apply to murine SGCs.^9^, ^51^ Interestingly, CNPase expression is reported to increase in rat SGCs following injury.^86^ Moreover, it was found that embryonic rat SGCs are able to divide and differentiate into other glial cell populations like oligodendrocytes, SCs and astrocytes in vitro.^87^ Another study reported that rat SGCs resemble SC precursors with the ability to myelinate embryonic axons in co‐cultures.^88^ Regarding murine SGCs, overexpression of Sox10 during embryogenesis was shown to transform SGCs towards an oligodendrocyte‐like phenotype, supporting the theory of SGCs being multipotent glial precursor cells with high plasticity.^89^ Within the present study, neither CNPase‐positive nor periaxin‐positive SGCs could be detected in Glb1^−/−^ or WT mice. These results indicate that murine SGCs do not upregulate markers for central or peripheral myelin in the course of G_M1_‐gangliosidosis.

### 4.7 Clinical deterioration in Glb1^−/−^ mice correlates with first signs of activation in dorsal root ganglia

Increased clinical scores, indicating a neurological impairment of Glb1^−/−^ mice, were noticed at 4 months of age (Figures 1 and 2). An increased expression of GFAP within SGCs was observed at the same time point (Figure 5). Therefore, it can be assumed that clinical deterioration in affected mice is accompanied by a reactive change in SGCs. Significant clinical differences between healthy WT and diseased Glb1^−/−^ mice were detected as early as 6 months of age in the grid‐walking and in the parachute test, accompanied by increased proliferation (6 months) and upregulation of nestin (8 months) in SGCs.

In conclusion, SGCs show phenotypical changes during G_M1_‐gangliosidosis characterized by upregulation of GFAP, increased proliferation and expression of the progenitor‐cell marker nestin. The obtained results point to the possibility of harnessing SGCs as a potential source of regulation, damage limitation and regeneration during the course of various nervous system diseases. However, further studies are needed to elucidate the function, reaction pattern and opportunities of differentiation of this unique cell population in vitro and in vivo.

## CONFLICT OF INTERESTS

The authors declare no conflict of interest.

## AUTHOR CONTRIBUTIONS

**Bei Huang:** Conceptualization (equal); Investigation (equal); Methodology (equal); Visualization (equal); Writing – original draft (equal); Writing – review & editing (equal). **Isabel Zdora:** Conceptualization (equal); Investigation (equal); Methodology (equal); Visualization (equal); Writing – original draft (equal); Writing – review & editing (equal). **Nicole de Buhr:** Investigation (equal); Visualization (equal); Writing – review & editing (equal). **Deborah Eikelberg:** Methodology (equal); Writing – review & editing (equal). **Wolfgang Baumgärtner:** Conceptualization (equal); Writing – original draft (equal); Writing – review & editing (equal). **Eva Leitzen:** Conceptualization (equal); Writing – original draft (equal); Writing – review & editing (equal).

## Supporting information

Figure S1‐S4

## Data Availability

Data were available on request from the authors.
