# Supplementary material for: Phenotypical changes of satellite glial cells in a murine model of GM1‐gangliosidosis
Source: J Cell Mol Med. 2021 Dec 7;26(2):527–39. doi: 10.1111/jcmm.17113 (PMC8743646; doi:10.1111/jcmm.17113)
Supplement: Supplementary file 1 — Figure S1‐S4 [file JCMM-26-527-s001.docx]

Supporting information

**
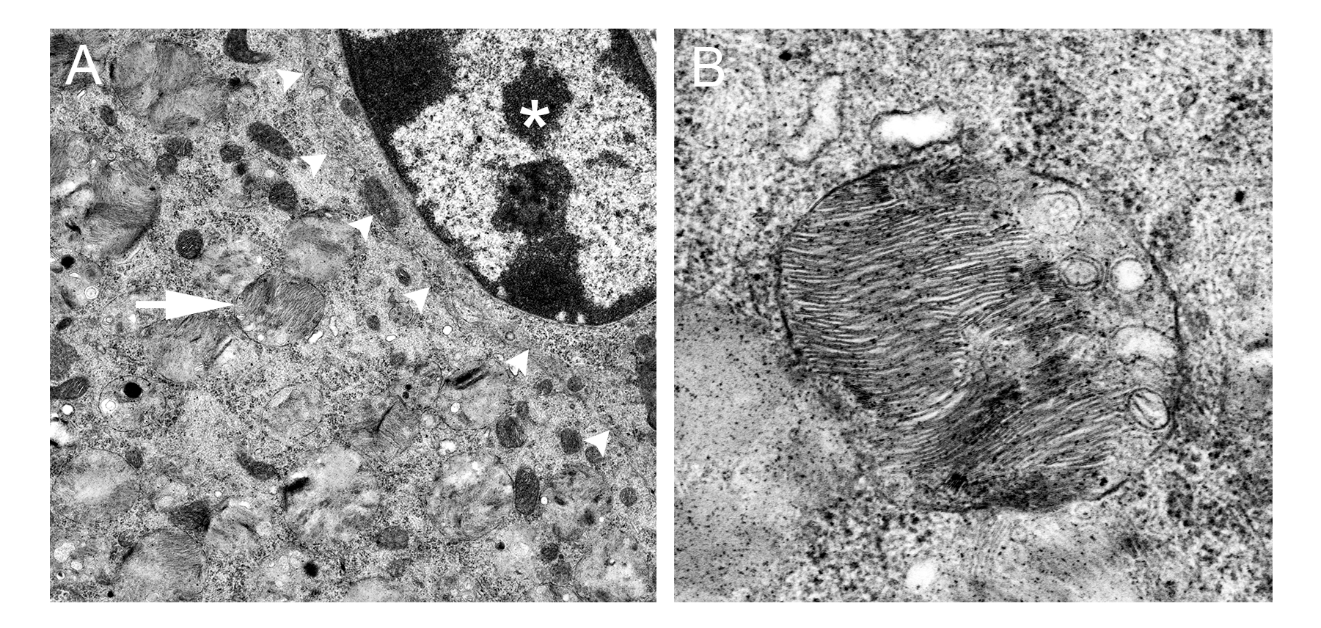
**

**Supplementary Figure 1:** (A, B) Transmission electron microscopic images of a dorsal root ganglion (DRG) of an 8-month-old Glb1^-/-^ mouse. (A) Satellite glial cell (SGC) nucleus (asterisk) located next to the cytoplasm of its ensheathed sensory neuron (cellular border between SGC and neuron indicated by arrow heads). Within the cytoplasm, lysosomal lamellated storage material is visible (arrow). (B) Higher magnification of the lysosomal storage material of the sensory neuron with a distinct lamellar structure.

**
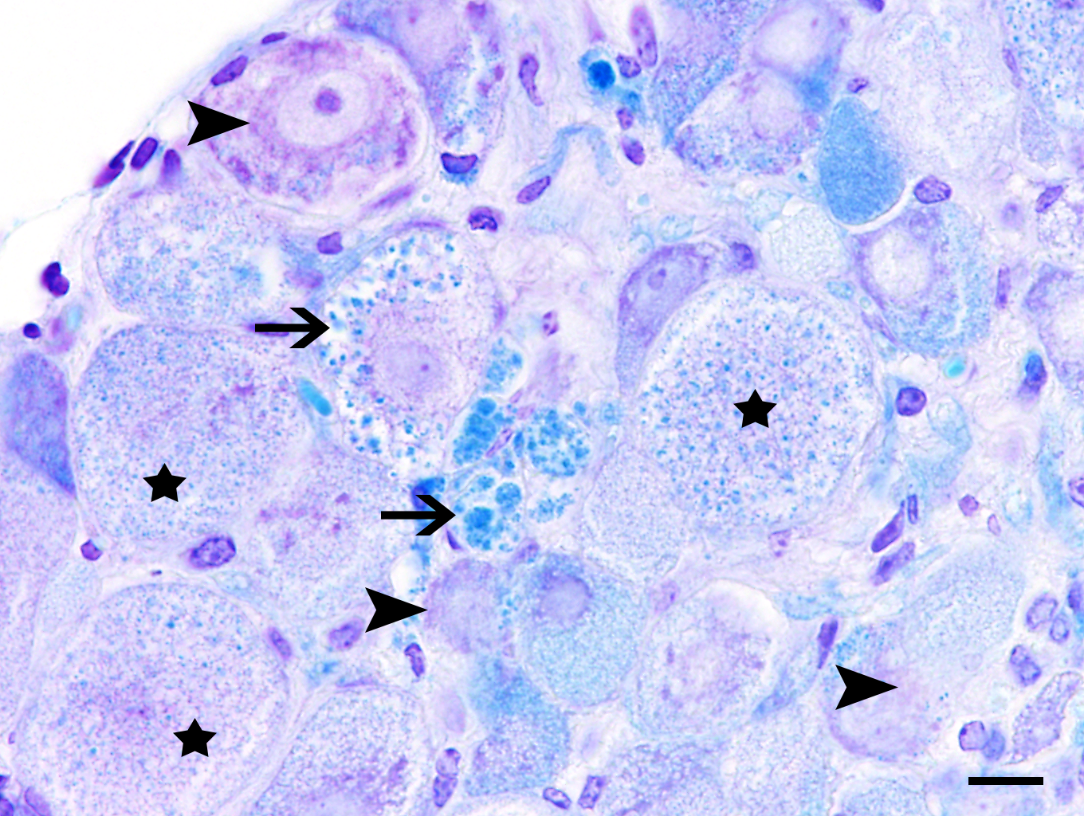
**

**Supplementary Figure 2:** Luxol fast blue (phospholipid staining) and cresyl violet stain of a dorsal root ganglion of an 8-months-old Glb1^-/-^ mouse. Neuronal cytoplasms show very prominent accumulation of blue, Luxol fast blue-positive, coarsely (arrows) and finely granulated (asterisks) storage material displacing and partly effacing the cresyl violet-positive Nissl substance (arrow heads). Scale bar, 10µm.

**
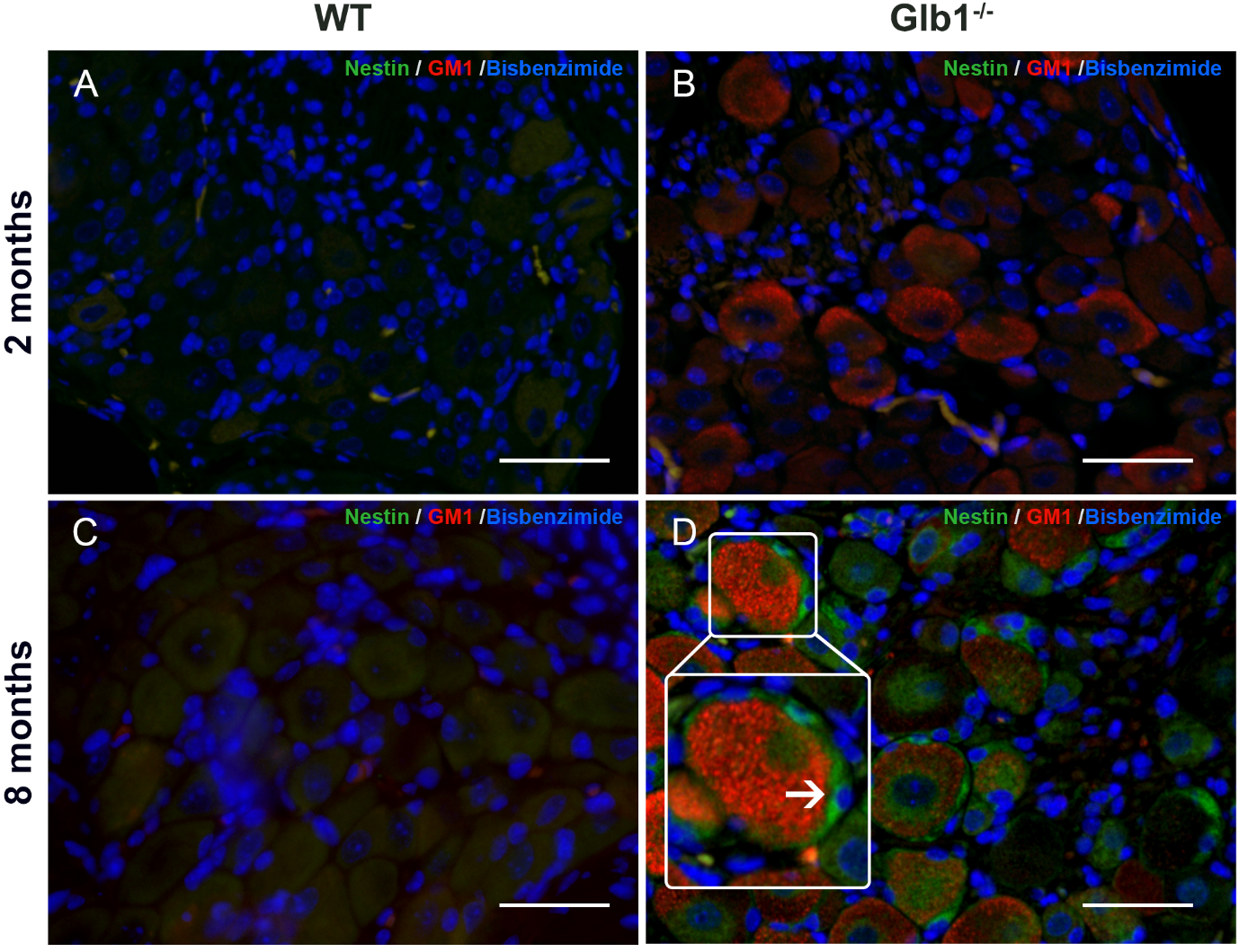
Supplementary Figure 3:** Representative images of immunofluorescence double staining of murine dorsal root ganglia (DRG) of 2 and 8-month-old Glb1^-/-^ and wild-type (WT) mice with the progenitor marker nestin (green) and GM1 (red). (**A, C**) No nestin expression and no ganglioside GM1 accumulation is visible in WT mice. (**B,** **D**) In 2-month-old Glb1^-/-^ mice, sensory neurons display storage of ganglioside GM1 but no nestin expression is visible in SGCs. In contrast, 8 months old Glb1^-/-^ mice exhibit nestin immunoreactivity in SGCs surrounding neurons with cytoplasmic accumulation of GM1-positive storage material (**D**; see insert for higher magnification; nestin-positive SGC indicated by arrow). Scale bar, 50 µm.

**
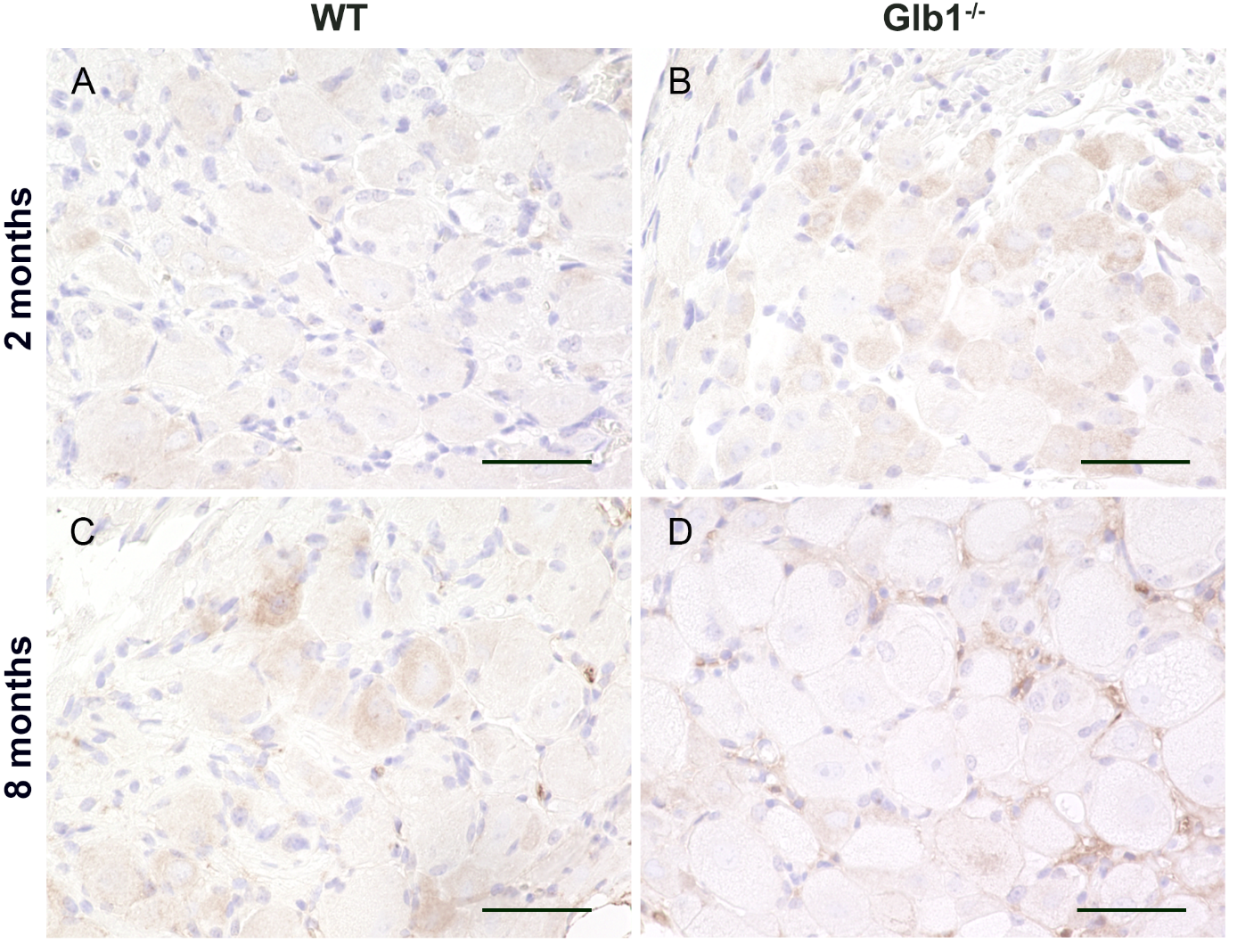
 Supplementary Figure 4:** Immunhistochemistry of dorsal root ganglia of 2- and 8-month-old wild-type (WT; **A**, **C**) and Glb1^-/-^ mice (**B**, **D**) for the neural progenitor marker doublecortin. None of the murine SGCs and sensory neurons show immunoreactivity for doublecortin at any time point. Scale bar, 50 µm.
